# Dual-Mode Microfluidic Workstation for Rapid Detection of Multiple Mycotoxins on Chip

**DOI:** 10.3390/foods14111928

**Published:** 2025-05-29

**Authors:** Binfeng Yin, Shiyu Zeng, Jun Liu, Rashid Muhammad, Zhuoao Jiang, Gang Tan, Qi Yang

**Affiliations:** 1 School of Mechanical Engineering, Yangzhou University, Yangzhou 225127, China; shiyuzeng315@foxmail.com (S.Z.); chemistwindow@gmail.com (R.M.); jza2422443660@outlook.com (Z.J.); gang.tan@foxmail.com (G.T.); qiyang223@foxmail.com (Q.Y.); 2 Suqian Product Quality Supervision and Inspection Institute, Suqian 223800, China; ljljljlj1982@163.com

**Keywords:** mycotoxins, dual-mode, microfluidic workstation, colorimetric, chemiluminescent (CL)

## Abstract

The assurance of food safety requires sensitive monitoring of multiple mycotoxins due to their severe impacts on the food industry and high health risks posed to consumers. Herein, we proposed a chemiluminescent/colorimetric dual-signal readout microfluidic method, incorporating a streptavidin-biotin-alkaline phosphatase (SA-Biotin-ALP) signal amplification system for the highly sensitive detection of Deoxynivalenol (DON), Ochratoxin A (OTA), and Aflatoxin B1 (AFB1). The indirect competitive enzyme-linked immunoassay (ic-ELISA) was integrated into microfluidic chip, resulting in sensitive detection ranges of DON in the range of 4–128 ng/mL, 2–64 ng/mL for OTA, and 0.2–6.4 ng/mL for AFB1, with the limit of detection (LOD) being 2.636 ng/mL, 1.492 ng/mL, and 0.131 ng/mL, respectively. Recovery rates in beer samples ranged from 91.93% to 109.31%. Furthermore, a dual-mode microfluidic workstation (DMMW) was developed to facilitate rapid, automated detection for these mycotoxins, simplifying the detection procedure, enhancing the detection efficiency, and reducing the requirement for specialized personnel, thus confirming significant potential for the rapid detection of mycotoxins in complex matrices such as beer.

## 1. Introduction

Mycotoxins are toxic metabolites produced by fungi, widely existing in rice, barley, wheat, and other food crops [1,2,3,4]. Mycotoxins produced by the metabolism of fungi usually pose serious health hazards to consumers. These toxic compounds are associated with a long list of adverse health effects, including nephrotoxicity, hepatotoxicity, gastrointestinal damage, immunosuppression, neurotoxicity, mutagenicity, carcinogenicity, and disruption to endocrine functions [5,6,7]. Studies have estimated that about 60–80% of global food crop production is contaminated with mycotoxins, with more than 30% of food and feed samples shown to be co-contaminated, leading to significant economic losses as a result of reductions in alcoholic beverage production and the increases in health care and regulatory costs [8,9,10]. To protect the health of consumers, it is necessary to develop a rapid and sensitive method for the detection of mycotoxins.

Currently, high-performance liquid chromatography (HPLC) and liquid chromatography–mass spectrometry (LC-MS/MS) are frequently used for the quantitative analysis of mycotoxins [11,12,13]. Although these conventional methods possessed high sensitivity and accuracy, their utilization is limited due to expensive instruments and tedious operating procedures [14,15,16]. One of the important trends in mycotoxin analysis is the development of simple and rapid analytical methods, so researchers are developing various methodologies for the analysis of mycotoxins [17,18,19,20]. Chen et al. developed a micropore resistance counting platform for multiplexed and ultrasensitive detection of mycotoxins with sensitivity in pg/mL [21]. Yang et al. proposed a N/O co-doping porous biomass carbon constructed electrochemical sensor for the universal and sensitive detection of mycotoxins, achieving detection ranges of 0.001–1000 pg/mL [22]. Shi et al. developed a programmable multichannel chemiluminescence immunoassay sensor for the quantification of mycotoxins with a detection limit of 16.32 μg/kg [23].

Microfluidic analysis platform technology has received considerable attention due to its miniaturization, integration, and automation [24,25,26]. Compared to traditional platforms or systems, microfluidic platforms can integrate diverse biochemical reactions into the microfluidic chip, which means less reagent cost, higher throughput, faster analysis, more precise control, and higher mobility [27,28,29]. This efficient platform provided a practical solution for the point-of-care testing (POCT) of multiple mycotoxins [30,31,32,33]. Various approaches have been integrated into microfluidic chips for rapid mycotoxin detection [34,35]. Wang et al. used a microfluidic-engineered portable microsphere sensors to successfully detect three fungal toxins: patulin, aflatoxin B1, and ochratoxin A [36]. Wu et al. reported the use of a comb-shaped microfluidic aptasensor for rapid and sensitive on-site simultaneous detection of aflatoxin B1 and deoxynivalenol [37]. However, the preparation of reagents, signal capture, and the storage of waste liquid in these methods usually require additional experimental instruments, indicating dependence on professional operators.

**Scheme 1:**
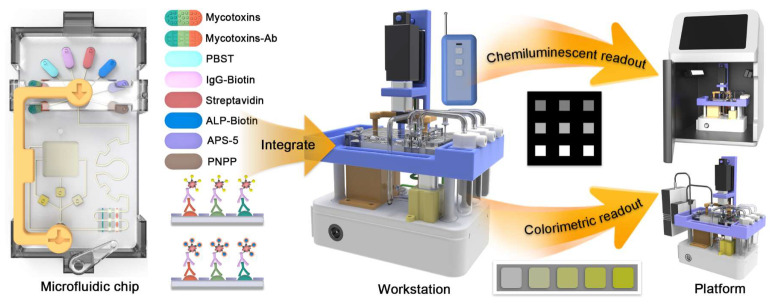
DMMW for detecting multiple mycotoxins.

Herein, we proposed a dual-mode chemiluminescent and colorimetric microfluidic workstation (DMMW), which is based on an indirect competitive immunoassay integrating streptavidin-biotin-alkaline phosphatase (SA-Biotin-ALP) signal amplification system for implementing rapid detection for DON, OTA, and AFB1 (Scheme 1). To achieve chemiluminescent/colorimetric dual signal readout, indirect competitive immunoassays were integrated in the reaction layer and three incubation reservoirs in the microfluidic chip for generating CL arrays and color signals. The efficient binding between antigen and antibody was achieved by a half-gear-shaped micromixer. A combination valve consisting of two valves controlled the reagents from eight reservoirs. Additionally, the DMMW integrating microfluidic chip was manufactured to automatically detect multiple mycotoxins, simplifying the operation, reducing the detection time, and improving the adaptability, which fulfills the requirement of rapid detection and exhibits great potential for detecting multiple mycotoxins in beer without advanced facilities.

## 2. Materials and Methods

### 2.1. Materials and Instruments

For the manufacture of the polydimethylsiloxane (PDMS) chip, Sylgard 184 silicone elastomer kit was obtained from Dow Corning Inc. (Midland, MI, USA). Silicon film was purchased from Shanghai Shentong Rubber and Plastic Products Co., Ltd. (Shanghai, China). The detecting reagents, including DON-ovalbumin (DON-OVA, >90%), OTA-ovalbumin (OTA-OVA, >90%), and AFB1-ovalbumin (AFB1-OVA, >90%) conjugates, antibodies (>90%) of DON, OTA, and AFB1, and DON, OTA and AFB1 standards (≥95%), were purchased from Zhejiang Zhunce Biotechnology Co., Ltd. (Hangzhou, China). Detection antibody conjugated with Biotin (IgG-Biotin, ≥95%), streptavidin (SA, ≥95%), and 4-nitrophenyl phosphate (PNPP, ≥98%) were purchased from Beijing Solarbio Technology Co., Ltd. (Beijing, China). Alkaline phosphatase conjugated with Biotin (Biotin-ALP, ≥95%) was obtained from Thermo Fisher Scientific (Waltham, MA, USA). Bovine Serum Albumin (BSA, >96%) fraction V powders were bought from Tianjin Kangyuan Biotechnology Co., Ltd. (Tianjin, China). PBS tablets and Tween-20 (≥98%) were obtained from Amresco (Framingham, MA, USA). Disodium [(4-chlorophenyl) sulfanyl] (10-methyl-9(10H)-acridinylidene) methyl phosphate (APS-5, >99%) was purchased from Changsha Xinlizhihe Technology Co., Ltd. (Changsha, China). Other reagents of analytical grade were obtained from Macklin Inc. (Shanghai, China).

The PDMS chip was fabricated using a YXIN-PRO LCD 3D printer from Yangzhou YIXIN 3D Technology Co., Ltd. (Yangzhou, China). For the microfluidic chip bonding process, a Putler plasma cleaner (Yantai, China) was employed. Microchannel images were obtained using a Shenzhen AOSVI TM28 metallographic microscope (Shenzhen, China). The colorimetric signals were detected by an XS11639 high-sensitivity fiber optic spectrometer from Shanghai Ruhai photoelectric Technology Co., Ltd. (Shanghai, China).

### 2.2. Design of Microfluidic Chip

The design of the microfluidic chip consists of PDMS layers, a reaction layer, a composite valve, and a bracket. The PDMS layers were divided into upper and lower layers. The upper layer was equipped with eight reservoirs for reagent deposit and three incubation reservoirs for the incubation of antigens. A semi-gear-shaped micromixer was incorporated on the upper layer to mix multiple liquids efficiently. The lower layer was equipped with a waste pool. The reaction layer was a silicon film coated with three antigens. The design of the composite valve was divided into valve 1, valve 2, and the connecting lug, controlling the flow of reagents. The design of the brackets included three parts to avoid excessive displacement of the valve and vibration of the chip, including the front bracket, the back bracket, and the valve bracket.

### 2.3. Fabrication of Microfluidic Chip

**Figure 1:**
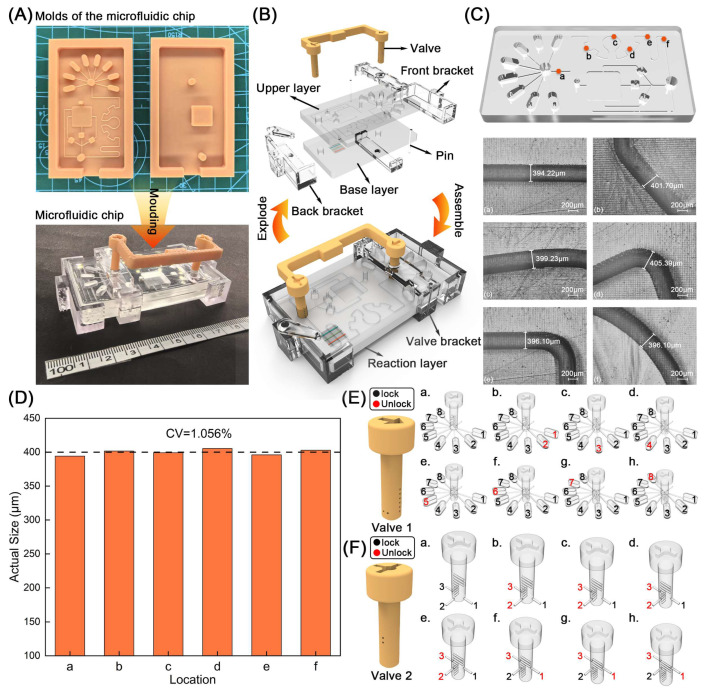
(**A**) The developed molds, the cured microfluidic chip, valve, and bracket. (**B**) Exploded and assembled diagrams of the microfluidic chip. (**C**) Photographs of microchannel (a–f) with the size ranging from 394.22 μm to 405.39 μm; (a–f) corresponding dimensions of six locations in the microchannel; (**D**) Quantitative analysis of the microchannel; (**E**) Valve 1 manipulation state for quantitative analysis. 1–8: Eight reservoirs of the microfluidic chip storing sample, capture antibody, PBST, IgG-Biotin, SA, ALP-Biotin, CL substrate, and PNPP, respectively. (**F**) Valve 2 manipulation state for quantitative analysis. 1–3: Three microchannels of the microfluidic chip.

To manufacture the microfluidic chip, we first fabricated the molds, composite valves, and brackets of the microfluidic chip through a light-curing 3D printer (Figure 1A). Then, the prepared mixture of PDMS with Sylgard 184 curing agent in a ratio of 10:1 was poured into the molds of the PDMS layers. The bubbles were removed through a vacuum drying oven. After removing air bubbles, the mold was placed in an oven at 65 °C for 3 h to cure the PDMS mixture. After the curing process, we used tweezers to remove the cured chip from the mold, removed the surface burrs, and punched 1.5 mm holes in the reservoirs and negative pressure port of the upper chip. Next, surface treatment of cured upper and lower chips was carried out using a plasma cleaner configured with parameters, including a power output of 200 W and an oxygen flow rate of 1.5 L/min. After that, the upper layer, reaction layer, and lower layer were tightly bonded for 60 s. Finally, the fabricated brackets and combination valve were assembled on the prepared PDMS layers to accomplish the fabrication of the microfluidic chip.

### 2.4. Lab on Microfluidic Chip

To realize simultaneous detection of multiple mycotoxins using fabricated microfluidic chips, we proposed a novel dual-mode chemiluminescent/colorimetric readout detection method for quantitative analysis of DON, OTA, and AFB1. Before detection, DON-OVA, OTA-OVA, and AFB1-OVA were encapsulated in three incubation reservoirs, respectively, and subsequently, 5% BSA was injected throughout the chip to block excess binding sites. First, we introduced 35 μL of different reagents into each of the eight reservoirs, including the sample, the capture antibody, PBST, IgG-Biotin, SA, Biotin-ALP, APS-5, and PNPP. Specifically, the samples included DON at concentrations of 4–128 ng/mL, OTA at concentrations of 2–64 ng/mL, and AFB1 at concentrations of 0.2–6.4 ng/mL. The corresponding capture antibodies, including DON-Ab, OTA-Ab, and AFB1-Ab, were set at concentrations of 5, 5, and 1.25 μg/mL, respectively. The negative pressure pump was connected to the negative pressure hole of the chip to drive the liquid to flow under the negative pressure. The combination valve was used to control the flow of liquid from the eight reservoirs. To start with, the valve controlled the simultaneous outflow of the sample and captured antibodies from the reservoirs. The fully mixed liquid through the micromixer flew into the reaction layer and three incubation reservoirs for the first immune reaction, and eventually into the waste pool. The valve then controlled the flow of PBST from the reservoir to clean the remaining antigens and antibodies in the channel. After cleaning, the valve continued to control the flow of the detection antibody IgG-Biotin into the reaction layer and incubation reservoirs for the second immune response. PBST was then introduced into the chip to clean the channel of excess detection antibodies. After cleaning, the valve-controlled SA in the reservoir flew into the reaction layer and incubation reservoirs for combination. PBST was then fed into the chip to clean the excess SA. Similarly, Biotin-ALP was then introduced into the reaction layer and incubation reservoirs for combination. PBST was then utilized again to wash away excess Biotin-ALP. In particular, after this cleaning, the valve controlled the flow of APS-5 into the reaction layer, bypassing the three incubation reservoirs to avoid cross-reaction, resulting in CL arrays. After the PBST cleaned the excess APS-5, the valve controlled the flow of PNPP into the three incubation reservoirs to produce yellow colorimetric signals with an absorbance of 405 nm. During the detection process, the CCD (charge-coupled device) camera would capture CL signals in real time. The colorimetric signals were detected by the fiber optic spectrometer.

### 2.5. Incubation of Antigens on Reaction Layer

To incubate DON-OVA, OTA-OVA, and AFB1-OVA on the reaction layer, a simple straight microchannel PDMS layer was prepared and covered with the silicon film. Multiple antigens, including DON-OVA, OTA-OVA, and AFB1-OVA, were then fed into three straight channels and incubated at 37 °C for 30 min. Next, PBST was introduced into the three straight channels for cleaning 5–7 times to remove excess antigens. Finally, the PDMS layer was removed with tweezers, and the incubation process was completed after the silicon film was completely dried.

### 2.6. Statistical Evaluation

To assess the detection performance of DMMW, key parameters, including the limit of detection (LOD), recovery rate, and relative standard deviation (RSD), were calculated and compared with other methods. The LOD was calculated based on a signal-to-noise ratio of 3:1, following the standard formula:

(1) $$LOD=\frac{3\sigma }{k}$$

where σ is the standard deviation of the five sets of blank values, and *k* is the slope of the standard curve.

Recovery was calculated following this formula:

(2) $$Recovery=\left(\frac{Q_{ds}-Q_{bs}}{Q_{s}}\right)\times 100\%$$

where *Q_s_* is the quantity of the spiked sample, *Q_bs_* is the quantity of basic sample, and *Q_ds_* is the detected quantity of the spiked sample.

The corresponding RSD was calculated following this formula:

(3) $$RSD=\frac{\sqrt{\frac{\sum_{i=1}^{n}{\left(x_{i}-\bar{x}\right)}^{2}}{n-1}}}{\bar{x}}\times 100\%$$

where $x_{i}$ is the calculated recovery, and $\bar{x}$ is the average recovery.

## 3. Results and Discussion

### 3.1. The Construction of Microfluidic Chip

The construction of a microfluidic chip primarily involves four components: PDMS layers, a reaction layer, a composite valve, and a set of brackets (Figure 1B). First, we modeled the PDMS layer through 3D modeling software. The PDMS layers were divided into upper and lower layers with sizes of 71 mm × 39 mm × 5 mm. The upper layer was equipped with eight 7 mm long, 3 mm wide, and 3 mm thick capsule-shaped reservoirs, holding 57 μL of liquid, and three square-shaped incubation reservoirs with a length of 3 mm, a width of 3 mm, and a thickness of 2 mm, storing 17 μL of reagents. Then, two valve holes with a diameter of 4 mm were arranged at the intersection of eight reservoirs and a three-pronged flow channel to match the valve to control the flow of reagents. In addition, on the upper layer, microchannels with a cross-section of 400 × 400 μm were utilized to connect the designed structure, including reservoirs, micromixer, valve holes, and a negative pressure hole. The lower layer of the PDMS was loaded with a 12 mm long, 12 mm wide, and 3.5 mm high waste pool, which can store 500 μL of reagents. The reaction layer was a silicon film 8 mm long, 8 mm wide, and 0.3 mm thick, coated with three antigens, including DON-OVA, OTA-OVA, and AFB1-OVA. The composite valve was divided into valve 1, valve 2, and the connecting lug, wherein the radius of the embedded part of valve 1 and valve 2 was set to 4.2 mm to ensure the interference fit and avoid the leakage of reagents when flowing through the valve. The connecting lug was designed between valve 1 and valve 2 to enable simultaneous actuation of both valves, arranged on the same side of the chip as much as possible to avoid interfering with the analysis of the detection signal in the PDMS layer (Figure 1B). The brackets included three parts to avoid excessive displacement of the valve and vibration of the chip, including the front bracket, the back bracket, and the valve bracket. In particular, the valve bracket and the back bracket were provided with process holes corresponding to the valve holes in the PDMS layer. Since the accuracy of the microchannel has a significant impact on the subsequent immune detection, we have characterized the upper-layer chip with complex microchannel structures through metallographic microscopy. In detail, we selected six locations (a–f) in the upper layer to characterize the differences between their measured dimensions and their design dimensions (Figure 1C). The width of the six channels ranges from 394.22 to 405.39 μm, with an error of 0.193–1.445%. The coefficient of variation (CV) of the channel is 1.056% (Figure 1D). These facts indicate the excellent processing technology of microfluidic chips.

While utilizing the composition valve to control the liquid, valve 1 would control eight reservoirs (1–8) storing different reagents, including the sample, capture antibody, PBST, IgG-Biotin, SA, Biotin-ALP, APS-5, and PNPP (Figure 1E). When valve 1 was in the first level (Figure 1(Ea)), the sample and the capture antibody in reservoirs 1 and 2 simultaneously passed through the valve and flowed into the microchannel. When the valve 1 was located at the second (Figure 1(Eb)), third (Figure 1(Ec)), fourth (Figure 1(Ed)), fifth (Figure 1(Ee)), and sixth levels (Figure 1(Ef)), PBST, IgG-Biotin, SA, Biotin-ALP, APS-5, and PNPP stored in respected reservoirs from 3 to 8 flew into subsequent microchannels for reaction. Meanwhile, valve 2, connected to valve 1, was also provided with the corresponding six-level channels to cooperate with valve 1 to realize the liquid distribution in the reservoirs by controlling the three microchannels in the chip (Figure 1F).

### 3.2. The Mixing Performance of Micromixer

**Figure 2:**
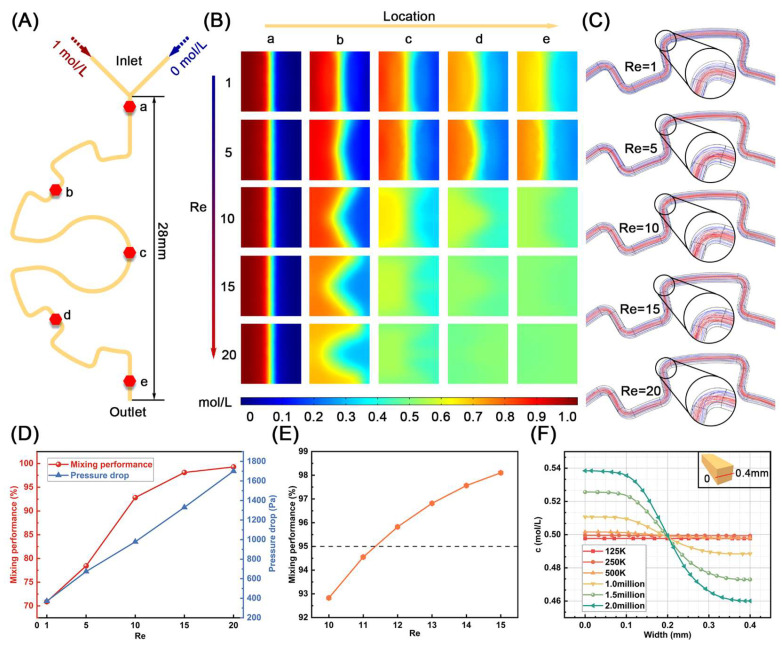
(**A**) Schematic drawing of the micromixer. (**B**) The mixing performance of each cross-section (a–e) at different Re. (a–e) Five locations of the micromixer in (**A**). (**C**) The streamlines in the micromixer with Re from 1 to 20. The mixing efficiency of the microchannel exit at Re ranges from (**D**) 1 to 20 and (**E**) 10 to 15. (**F**) Grid independence verification of the micromixer.

The mixing efficiency in microfluidic chips significantly influences antibody–antigen binding efficiency, thereby affecting the sensitivity of chemiluminescence detection. Therefore, optimizing micromixers to enhance mixing performance is critically important. Based on this background, we designed a semi-geared micromixer to improve the fluid mixing behavior in the chip (Figure 2A). To evaluate the mixing efficiency of the micromixer, we utilized COMSOL 5.6 software to simulate the mixing performance. Specifically, five locations (a–e) on the micromixer were identified to study the mixing behavior at different Reynolds numbers (Re). As shown in Figure 2B, the mixing efficiency increases as the position gets closer to the exit, indicating improved fluid mixing behavior. With the increase in Re, except for the position near the entrance, the concentration dividing line at the other positions gradually changed from the initially obvious straight line to a fuzzy curve type, which also indicated that the fluid mixing efficiency in the micromixer was improved. Interestingly, the streamlines in the micromixer also indicated this fact. As shown in Figure 2C, when Re = 1, there was no bending and disturbance. When Re = 5, 10, the streamlines began to bend, but the disturbance was still small at this time, and when Re > 10, the streamlines began to bend significantly and create eddy currents to accelerate the mixing. When focusing on the mixing performance at the outlet, the mixing efficiency has exceeded 95% at a Reynolds number of 15, which indicates excellent mixing (Figure 2D) [38]. However, a higher Re often means that the flow channel needs to withstand greater pressure, which is more likely to cause channel breakage and even leakage. To achieve optimum mixing while minimizing the pressure brought by high flow rates on microchannels, Re was further studied from 10 to 15 cases. When Re = 12, the mixing efficiency exceeds 95%, so Re = 12 was chosen as the final flow parameter (Figure 2E). Moreover, since the number of grids deployed on the micromixer model during the simulation process affected the simulation results, grid independence verification was carried out. In detail, one-dimensional cut lines connected by midpoints on the left and right sides of the section at the exit were selected to study the concentration distribution. The results showed that when the number of grids was 1.5 million, the maximum error on the transversal line was less than 2.5%, which meets the actual demand (Figure 2F). Therefore, the number of grids was chosen to be 1.5 million.

### 3.3. The Microfluidic Chip for the Detection

To detect small molecules, including DON, OTA, and AFB1, it is necessary to employ an indirect competitive enzyme-linked immunosorbent assay (ic-ELISA) rather than direct immunization. However, when detecting biomarkers based on ic-ELISA, as the concentration of the substance increases, the detection signal becomes weaker, forcing reduced sensitivity and increased requirements for laboratory equipment. Therefore, streptavidin-biotin-alkaline phosphatase (SA-Biotin-ALP) signal amplification system was integrated on the microfluidic chip to improve stability and sensitivity.

**Figure 3:**
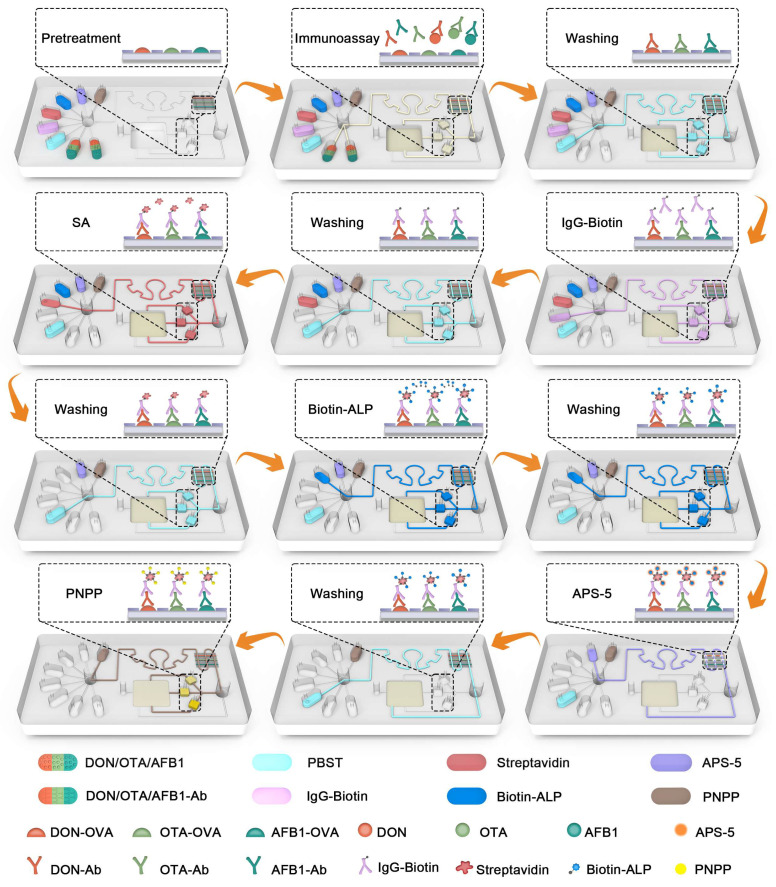
Schematic diagram of the microfluidic chip for DON, OTA, and AFB1 detection by the chemiluminescent and colorimetric immunoassay.

In Figure 3, eight reagents, including the sample, capture antibody, PBST, IgG-Biotin, SA, ALP-Biotin, APS-5, and PNPP, were introduced into the chip. First, the sample with DON, OTA, and AFB1 and the capture antibodies DON-Ab, OTA-Ab, and AFB1-Ab were introduced simultaneously into the reaction layer and three incubation reservoirs. Antigens in the sample competed with on-chip antigens DON-OVA, OTA-OVA, and AFB1-OVA for the capture antibodies. Antibodies bound to on-chip antigens remained in the reaction layer and incubation reservoirs. Biotin-labeled detection antibodies of IgG-Biotin were then introduced into the reaction layer and incubation reservoirs to bind specifically to capture antibodies. SA was then introduced into the reaction layer and incubation reservoirs to combine with the biotin on detection antibodies. Biotin-ALP was then introduced to bond with the SA on the detection antibody to form IgG-Biotin-SA- (Biotin-ALP) _n_ conjugate. Next, APS-5 was introduced into the reaction layer to react with ALP to produce CL arrays and bypassed the incubation reservoirs to avoid cross-reactions. Finally, PNPP flew into the incubation reservoirs and reacted with ALP to produce yellow signals with an absorbance of 405 nm. Significantly, each key detecting procedure in the microfluidic chip was connected to 5–7 times PBST cleaning to avoid cross-reaction and affect the actual test. The detected signals, including the CL signal and color signal, were negatively correlated with the concentration of mycotoxins.

### 3.4. The Establishment of DMMW

To realize the remote control and automatic rapid detection of multiple mycotoxins, based on the manufactured microfluidic chip, we developed a dual-mode chemiluminescent and colorimetric microfluidic workstation (DMMW) integrating with a CCD imaging system, simplifying the detection steps, improving adaptability, and reducing dependence on professionals.

**Figure 4:**
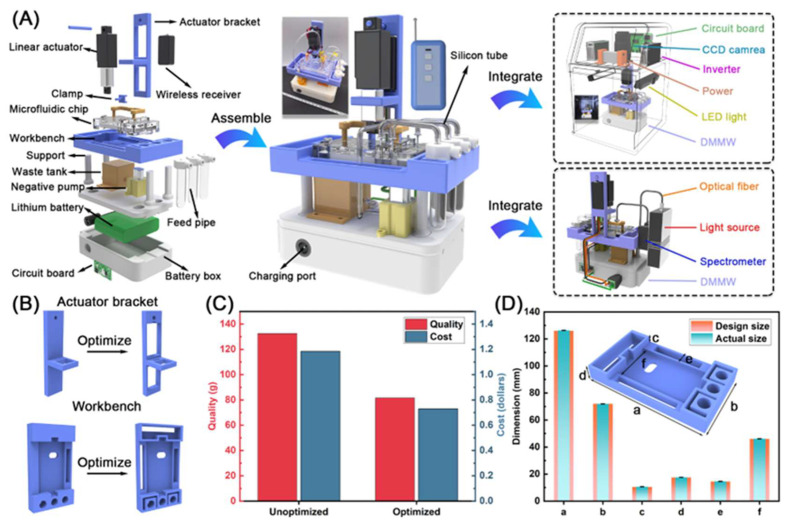
(**A**) The exploded and assembled DMMW integrated with the CCD system and fiber optic spectrometer. (**B**) Optimization of key parts (actuator bracket and workbench). (**C**) The comparison of quality and cost between unoptimized and optimized key parts. (**D**) The comparison of actual and design dimensions of the workbench.

The design of DMMW was composed of a microfluidic chip, frame, reagent supply module, power supply module, and drive module. The frame manufactured by 3D printing contained an actuator bracket, workbench, support, and battery box (Figure 4A). The reagent supply module consisted of three 2 mL liquid supply tubes, storing PBST, APS-5, and PNPP, respectively. The power supply module was a 12 V lithium battery with a DC connector and a charging connector; the driving module consisted of a wireless receiver, a linear actuator, a clamp, a circuit board, a negative pressure pump, and a waste tank. When the microfluidic chip was installed in the DMMW, the linear actuator was connected to the combination valve on the chip through the claw to control the valve automatically. In addition, silicone tubes and needles were used for the connection of the microfluidic chip, negative pressure pump, and reagent supply module. The DMMW was then placed into a CCD camera system to capture the CL signal. The optical density (OD_405nm_) was detected using the fiber optic spectrometer. The remote control was connected to the wireless receiver to regulate the vertical displacement of the linear actuator and the on-off operation of the negative pressure pump. Upon completion of one detection, the microfluidic chip will be replaced to prepare for the next detection. To facilitate the layout of wires and silicone tubes in DMMW and reduce the overall manufacturing cost as much as possible, two key parts in DMMW were selected, including the actuator bracket and workbench for lightweight design. Specifically, the original solid parts of these two parts have been hollowed out to the thinnest position where the wall thickness was greater than 3 mm to ensure sufficient strength. The manufacturing cost of the two parts before and after optimization was calculated with reference to the following formula:

(4) C=ρ·V·n

where ρ is the density of resin, which is 1.15 g/cm^3^ here; V is the volume of the two parts; and *n* is the unit price of the resin, which is 8.84 dollars/kg here.

Compared with the solid structure, based on no fracture under full load, the optimized two-part cost decreased by 38.4%. Additionally, to evaluate the machining accuracy of DMMW, six positions (a–f) on the workbench were selected for measurement to evaluate the accuracy of 3D-printing frame processing. As shown in Figure 4D, the machining error varies between 0.273 and 1.39%, indicating excellent machining accuracy.

### 3.5. Detection Performance

**Figure 5:**
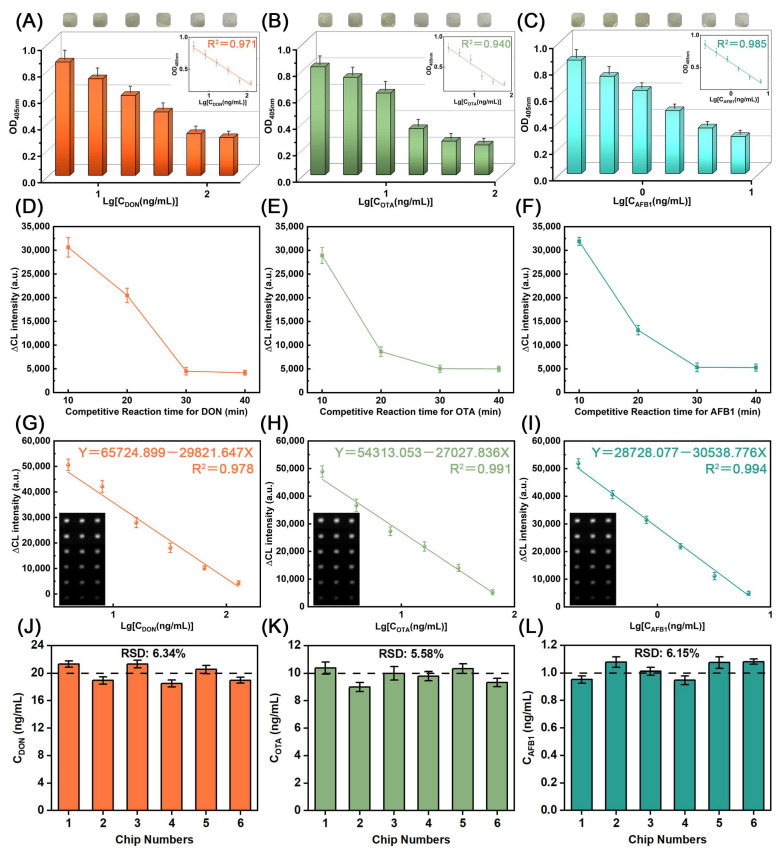
OD_405nm_ of (**A**) DON, (**B**) OTA, and (**C**) AFB1 at different concentrations. Optimization of the competitive reaction time for detecting (**D**) DON, (**E**) OTA, and (**F**) AFB1. Liner range of (**G**) DON, (**H**) OTA, and (**I**) AFB1 by CL intensity. Reproducibility of DON (**J**), OTA (**K**), and AFB1 (**L**).

To realize sensitively detecting DON, OTA, and AFB1 by DMMW, the concentrations of the coated antigens DON-OVA, OA-OVA, and AFB1-OVA were set to 10, 5, and 2.5 μg/mL, respectively; the concentrations of the corresponding capture antibodies DON-Ab, OTA-Ab, and AFB1-Ab were set to 5, 5, and 1.25 μg/mL [39]. IgG-Biotin was diluted 1000 times. The mass ratio of SA and Biotin-ALP was set to 1:4 [40]. First, we evaluate the practicality of this colorimetric method for rapid detection (Figure 5A–C). As the concentrations of the three mycotoxins increased, visible color changes occurred in the incubation reservoirs, observable to the naked eye, proving that the colorimetric method can be applied to the qualitative analysis of multiple mycotoxins. Furthermore, when the optical density of the liquids in these incubation reservoirs was measured using a portable spectrometer at 405 nm, a strong linear correlation emerged between OD_405nm_ and mycotoxin concentrations, with the regression coefficients (R^2^) of 0.971, 0.940, and 0.985. These facts indicate that the colorimetric method is sensitive enough for the quantitative analysis of multiple mycotoxins. Then, the chemiluminescent method was also employed for detecting three mycotoxins. Since the competitive reaction time in the immune response has a significant impact on the sensitivity of the assay, the competitive response time for mycotoxins was optimized. As shown in Figure 5D–F, the CL signal intensity gradually weakened and stabilized at 30 min as the competitive reaction time became longer. Therefore, 30 min was selected as the final incubation time. Based on optimized conditions, 4–128 ng/mL DON, 2–64 ng/mL OTA, and 0.2–6.4 ng/mL AFB1 were detected, respectively. As shown in Figure 5G–I, the relative values of the concentrations of the three mycotoxins showed obvious linear relationships with the corresponding CL intensity, with LODs of 2.636 ng/mL, 1.492 ng/mL, and 0.131 ng/mL, according to the three-time signal-to-noise ratio. Based on the linear equation obtained, we verified the accuracy of the method by using DMMW to detect specific concentrations of multiple mycotoxins on six microfluidic chips. Specifically, 20 ng/mL DON samples, 10 ng/mL OTA samples, and 1.0 ng/mL AFB1 samples were measured. The relative standard deviation (RSD) of the measured samples varied from 5.58% to 6.34% (Figure 5J–L), indicating the high accuracy of the method. These facts indicated that the proposed dual-mode chemiluminescent/colorimetric method can achieve sensitive detection of DON, OTA, and AFB1. Based on the optimized condition, DMMW was utilized to detect beer samples spiked with mycotoxins. As shown in Table 1, the recovery of DON ranged from 91.93% to 108.04% with the RSD of 6.43–10.08%. Similarly, the recovery of OTA was 94.23–109.31% with the RSD of 6.17–10.78%. The recovery of AFB1 was 93.47–105.38% with the RSD of 7.24–11.82%. Compared with other methods (Table 2), the dual chemiluminescent/colorimetric detection method exhibited great performance in LOD, RSD, and recovery. These results confirm the method’s validity for real sample analysis.

## 4. Conclusions

In summary, a DMMW integrating the SA-B-ALP signal amplification system was proposed for the rapid detection of DON, OTA, and AFB1 in beer samples. Based on the dual-mode chemiluminescent/colorimetric signal readout, the developed DMMW enabled the quantitative and simultaneous analysis of multiple mycotoxins, including DON ranging from 4 to 128 ng/mL, OTA ranging from 2 to 64 ng/mL, and AFB1 ranging from 0.2 to 6.4 ng/mL. Compared with other methods, DMMW integrating dual-signal readout greatly reduced the interference of environmental factors with strong adaptability. Furthermore, in real samples, DMMW exhibited high and acceptable recoveries. These results confirmed the potential of DMMW as an effective platform for environmental monitoring, medical diagnosis, and food safety detection. The demands for standardization, integration, and batch quantity also presented new challenges for implementing DMMW in real-world settings.

## Figures and Tables

**Table 1:** Recovery of DON, OTA, and AFB1 in beer samples.

DON			OTA			AFB1		
Spiked Value (ng/mL)	Recovery (%)	RSD (%)	Spiked Value (ng/mL)	Recovery (%)	RSD (%)	Spiked Value (ng/mL)	Recovery (%)	RSD (%)
5	104.46	7.01	0.25	94.26	8.19	2.5	103.45	11.82
10	92.98	6.43	0.5	106.34	6.17	5	105.38	7.68
20	105.35	6.57	1	94.23	10.78	10	96.46	8.23
40	91.93	10.08	2	104.29	7.74	20	93.47	7.24
80	108.04	8.07	4	109.31	6.31	40	95.82	6.59

**Table 2:** Comparison of the limit of detection (LOD), real analysis, signal form, and recovery rates between the DMMW described in this article and other reported platforms for detecting mycotoxins.

Platform	LFIC	cFMEIS	UCNPs-mICA	SBCI	Ce-MOF@MIP	DMMW
Mycotoxins	DON AFB1 ZEA	AFB1 OTA DON ZEA	DON AFB1 ZEA	ZEA	AFB1	DON OTA AFB1
LOD	73 ng/mL 45 ng/mL 43 ng/mL	0.032 μg/kg 0.141. μg/kg 0.097 μg/kg 0.376 μg/kg	0.25 ng/mL 0.05 ng/mL 0.1 ng/mL	3 μg/kg	0.25 ng/mL	2.636 ng/mL 1.492 ng/mL 0.131 ng/mL
Real sample	Oat, cornmeal, millet	Wheat flour	Corn, wheat	Corn	Peanuts, chicken feed, corn	Beer
Signal form	Photothermal	Fluorescence	Fluorescence	SERS	Colorimetry	Chemiluminescence, colorimetry
Recovery (%)	78.6–112.4	81.6–120	85.25–114.86	90.58–105.29	95.1–109.4	91.93–109.31
RSD (%)		2.80–7.20		<6.00	0.53–1.71	6.43–11.82
References	[41]	[42]	[43]	[44]	[45]	This work

## Data Availability

The original contributions presented in this study are included in the article. Further inquiries can be directed to the corresponding author.

## Abbreviations

The following abbreviations are used in this manuscript:

DMMW	Dual-Mode microfluidic Workstation
SA-Biotin-ALP	Streptavidin-biotin-alkaline phosphatase
DON	Deoxynivalenol
OTA	Ochratoxin A
AFB1	Aflatoxin B1
ic-ELISA	Indirect competitive enzyme-linked immunoassay
LOD	limit of detection
CL	Chemiluminescent
HPLC	High-performance liquid chromatography
LC-MS	Liquid chromatography–mass spectrometry
POCT	Point of care testing
PDMS	Polydimethylsiloxane
OVA	Ovalbumin
IgG-Biotin	Detection antibody conjugated with Biotin
SA	Streptavidin
PNPP	4-nitrophenyl phosphate
Biotin-ALP	Alkaline phosphatase conjugated with Biotin
BSA	Bovine Serum Albumin
APS-5	Disodium [(4-chlorophenyl) sulfanyl] (10-methyl-9(10H)-acridinylidene) methyl phosphate
Ab	Antibody
RSD	Relative standard deviation
CV	Coefficient of variation
Re	Reynolds numbers
CCD	Charge-coupled Device
OD	Optical density

## References

[1] Hou Y.J., Jia B.Y., Sheng P., Liao X.F., Shi L.C., Fang L., Zhou L.D., Kong W.J. (2022). Aptasensors for mycotoxins in foods: Recent advances and future trends. Compr. Rev. Food Sci. Food Saf..

[2] Bräse S., Encinas A., Keck J., Nising C.F. (2009). Chemistry and Biology of Mycotoxins and Related Fungal Metabolites. Chem. Rev..

[3] Anater A., Manyes L., Meca G., Ferrer E., Luciano F.B., Pimpao C.T., Font G. (2016). Mycotoxins and their consequences in aquaculture: A review. Aquaculture.

[4] Hussein H.S., Brasel J.M. (2001). Toxicity, metabolism, and impact of mycotoxins on humans and animals. Toxicology.

[5] Pitt J.I., Miller J.D. (2017). A Concise History of Mycotoxin Research. J. Agric. Food Chem..

[6] Yin S.T., Liu X.Y., Fan L.H., Hu H.B. (2018). Mechanisms of cell death induction by food-borne mycotoxins. Crit. Rev. Food Sci. Nutr..

[7] Awuchi C.G., Ondari E.N., Nwozo S., Odongo G.A., Eseoghene I.J., Twinomuhwezi H., Ogbonna C.U., Upadhyay A.K., Adeleye A.O., Okpala C.O.R. (2022). Mycotoxins’ Toxicological Mechanisms Involving Humans, Livestock and Their Associated Health Concerns: A Review. Toxins.

[8] Wang X., Bouzembrak Y., Lansink A., Van der Fels-Klerx H.J. (2022). Designing a monitoring program for aflatoxin B1 in feed products using machine learning. Npj Sci. Food.

[9] Pinotti L., Ottoboni M., Giromini C., Dell’Orto V., Cheli F. (2016). Mycotoxin Contamination in the EU Feed Supply Chain: A Focus on Cereal Byproducts. Toxins.

[10] Eskola M., Kos G., Elliott C.T., Hajslová J., Mayar S., Krska R. (2020). Worldwide contamination of food-crops with mycotoxins: Validity of the widely cited ‘FAO estimate’ of 25%. Crit. Rev. Food Sci. Nutr..

[11] Zhou W., Wieczorek M.N., Pawliszyn J. (2023). High throughput and automated solid-phase microextraction and determination by liquid chromatography-mass spectrometry for the analysis of mycotoxins in beer. Food Chem..

[12] Hickert S., Gerding J., Ncube E., Hübner F., Flett B., Cramer B., Humpf H.U. (2015). A new approach using micro HPLC-MS/MS for multi-mycotoxin analysis in maize samples. Mycotoxin Res..

[13] Wang Y.L., Chai T.J., Lu G.Z., Quan C.S., Duan H.Y., Yao M.L., Zucker B.A., Schlenker G. (2008). Simultaneous detection of airborne Aflatoxin, Ochratoxin and Zearalenone in a poultry house by immunoaffinity clean-up and high-performance liquid chromatography. Environ. Res..

[14] Zhu W.R., Li L.B., Zhou Z., Yang X.D., Hao N., Guo Y.S., Wang K. (2020). A colorimetric biosensor for simultaneous ochratoxin A and aflatoxins B1 detection in agricultural products. Food Chem..

[15] Chen Y.Q., Chen Q., Han M.M., Zhou J.Y., Gong L., Niu Y.M., Zhang Y., He L.D., Zhang L.Y. (2016). Development and optimization of a multiplex lateral flow immunoassay for the simultaneous determination of three mycotoxins in corn, rice and peanut. Food Chem..

[16] Maragos C.M., Busman M. (2010). Rapid and advanced tools for mycotoxin analysis: A review. Food Addit. Contam. Part A-Chem..

[17] Wang C.L., Sha T., Lu J.S., Guan Y.F., Geng X.H. (2024). A Miniaturized and Highly Sensitive “Windmill” Three-Channel Fluorescence Detector for Simultaneous Detection of Various Mycotoxins. Anal. Chem..

[18] Adunphatcharaphon S., Kolawole O., Sooksimuang T., Panchan W., Wasuthep W., Petdum A., Pichayawaytin G., Jintamethasawat R., Doljirapisit N., Somboonkaew A. (2024). A multiplex microarray lateral flow immunoassay device for simultaneous determination of five mycotoxins in rice. Npj Sci. Food.

[19] Yang Q., Xiong J.H., Duan L.Y., Chen S.D., Peng Z.J., Liao X.N., Ning Z.Q., Wang D. (2024). Polydopamine@ZIFs with enhanced electrochemiluminescence quenching performance for mycotoxin detection. Food Chem..

[20] Zhang Y., Zhao C.P., Picchetti P., Zheng K.Y., Zhang X.A., Wu Y.L., Shen Y., De Cola L., Shi J.Y., Guo Z.M. (2024). Quantitative SERS sensor for mycotoxins with extraction and identification function. Food Chem..

[21] Li L.T., Wang M.J., Dong Y.Z., Yu D.Y., Chen Y.P. (2025). Micropore Resistance Counting Platform for Multiplexed and Ultrasensitive Detection of Mycotoxins and Biomarkers. ACS Nano.

[22] Yang W., Li X.N., Zhang M.J., Wang Q., Wang Y.J., Yu S.S., Dang R.H., Wang X.R., Yang Z., Fan S.H. (2025). N/O co-doping porous biomass carbon constructed electrochemical sensor for universal and sensitive detection to mycotoxins. Food Chem..

[23] Shi Y.K., Lang H.Y., Ye J., Xuan Z.H., Ni B.X., Xie Y.L., Liu H.M., Wang S.X. (2025). A programmable multichannel chemiluminescence immunoassay sensor for automatic quantification of the total amount of fumonisin B1, B2 and B3. Food Chem..

[24] Yin B.F., Zhu H.Y., Zeng S.Y., Sohan A., Wan X.H., Liu J., Zhang P., Lin X.D. (2024). Chip-based automated equipment for dual-mode point-of-care testing foodborne pathogens. Biosens. Bioelectron..

[25] Zeng S.Y., Zhu H.Y., Sohan A., Liu J., Wan X.H., Lin X.D., Yin B.F. (2024). A remote-controlled portable workstation for highly sensitive and real-time chemiluminescent detection of cadmium. Food Chem..

[26] Lin X.D., Wu H.T., Zeng S.Y., Peng T., Zhang P., Wan X.H., Lang Y.H., Zhang B., Jia Y.W., Shen R. (2023). A self-designed device integrated with a Fermat spiral microfluidic chip for ratiometric and automated point-of-care testing of anthrax biomarker in real samples. Biosens. Bioelectron..

[27] Jiang Z.A., Zhuang Y., Guo S.T., Sohan A., Yin B.F. (2023). Advances in Microfluidics Techniques for Rapid Detection of Pesticide Residues in Food. Foods.

[28] El-Ali J., Sorger P.K., Jensen K.F. (2006). Cells on chips. Nature.

[29] Yin B.F., Wan X.H., Qian C.C., Sohan A., Zhou T., Yue W.K. (2021). Enzyme Method-Based Microfluidic Chip for the Rapid Detection of Copper Ions. Micromachines.

[30] Soares R.R.G., Santos D.R., Pinto I.F., Azevedo A.M., Aires-Barros M.R., Chu V., Conde J.P. (2018). Multiplexed microfluidic fluorescence immunoassay with photodiode array signal acquisition for sub-minute and point-of-need detection of mycotoxins. Lab. Chip.

[31] Soares R.R.G., Santos D.R., Chu V., Azevedo A.M., Aires-Barros M.R., Conde J.P. (2017). A point-of-use microfluidic device with integrated photodetector array for immunoassay multiplexing: Detection of a panel of mycotoxins in multiple samples. Biosens. Bioelectron..

[32] Chen Y.Q., Meng X.R., Zhu Y.Z., Shen M.J., Lu Y., Cheng J., Xu Y.C. (2018). Rapid detection of four mycotoxins in corn using a microfluidics and microarray-based immunoassay system. Talanta.

[33] Lin X.D., Cao Z.Y., Zeng S.Y., Zhu H.Y., Zhai K.R., Yin B.F., Zhang C., Peng T., Cheng T., Zhang B. (2025). Portable dual-mode microfluidic sensor for rapid and sensitive detection of DPA on chip. Adv. Compos. Hybrid Mater..

[34] Xiang X.R., Song M.H., Xu X.W., Lu J.R., Chen Y.Y., Chen S.H., He Y.L., Shang Y.T. (2023). Microfluidic Biosensor Integrated with Signal Transduction and Enhancement Mechanism for Ultrasensitive Noncompetitive Assay of Multiple Mycotoxins. Anal. Chem..

[35] Hua M.Z., Liu J.X., Roopesh M.S., Lu X.N. (2024). Microfluidic Optical Aptasensor for Small Molecules Based on Analyte-Tuned Growth of Gold Nanoseeds and Machine Learning-Enhanced Spectrum Analysis: Rapid Detection of Mycotoxins. ACS Sens..

[36] Wang X.R., Xing G.W., Wu Z.N., Lin H.F., Lin Y.N., Lin J.X., Xie Y.S., Liao W.J., Lin L. (2025). Microfluidic-engineered portable microsphere sensors for multi-mycotoxins detection. Chem. Eng. J..

[37] Wu J.L., Yuan H.J., Yang Y.H., Yang P., Yan X.X., Mu Y., Jin Q.H., Yang P.H., Gao W.L. (2025). A comb-shaped microfluidic aptasensor for rapid and sensitive on-site simultaneous detection of aflatoxin B1 and deoxynivalenol. Food Chem..

[38] Zeng S.Y., Sun X.C., Wan X.H., Qian C.C., Yue W.K., Sohan A., Lin X.D., Yin B.F. (2022). A cascade Fermat spiral microfluidic mixer chip for accurate detection and logic discrimination of cancer cells. Analyst.

[39] Liu J., Zeng S.Y., Zhu H.Y., Wan X.H., Sohan A., Yin B.F. (2024). A Portable Automated Microfluidic Platform for Point-of-Care Testing for Multiple Mycotoxins in Wine. Foods.

[40] Wu J., Chen Y.P., Yang M.Z., Wang Y., Zhang C., Yang M., Sun J.S., Xie M.X., Jiang X.Y. (2017). Streptavidin-biotin-peroxidase nanocomplex-amplified microfluidics immunoassays for simultaneous detection of inflammatory biomarkers. Anal. Chim. Acta.

[41] Huang Q., Yang H.J., Wang W.L., Zhang Y. (2023). Multi-target photothermal immunochromatography for simultaneous detection of three mycotoxins in foods. Anal. Chim. Acta.

[42] Lin X.Q., Ge R., Wei J., Jiao T.H., Chen Q.M., Oyama M., Chen Q.S., Chen X.M. (2024). Magnetic-encoded fluorescent nanospheres-based competitive immunoassay for near-simultaneous detection of four mycotoxins in wheat. Food Chem..

[43] Shen Y.H., Chen Y.Y., Wang H.F., Zhao X.L., Lu H.X., Zhu J.L. (2024). Immunochromatographic assay integrated smartphone-based device for simultaneous detection of multiple mycotoxins using core-shell up-conversion nanoparticles. Sens. Actuator B-Chem..

[44] Yin L.M., You T.Y., Arslan M., El-Seedi H.R., Guo Z.M., Zou X.B., Cai J.R. (2023). Dual-layers Raman reporter-tagged Au@Ag combined with core-satellite assemblies for SERS detection of Zearalenone. Food Chem..

[45] Damphathik C., Prakobkij A., Jarujamrus P., Boonmak J., Suebphanpho J., Bunkoed O., Samphao A. (2025). Colorimetric sensor comprising metal-organic frameworks and molecularly imprinted polymers for aflatoxin B1 detection in agricultural commodities. Food Chem..

